# Influence of Mineral Particle Films and Biomaterials on Guava Fruits and Implications for the Oviposition of *Anastrepha obliqua* (Macquart) (Diptera: Tephritidae)

**DOI:** 10.3390/insects12050373

**Published:** 2021-04-21

**Authors:** Daniela Ribeiro da Costa, Suzany Aguiar Leite, Mateus Pereira dos Santos, Beatriz Sousa Coelho, Aldenise Alves Moreira, Carlos Alberto Domingues da Silva, Iara Sordi Joachim-Bravo, Maria Aparecida Castellani

**Affiliations:** 1 Department of Crop Science and Animal Science, State University of Southwestern Bahia, Vitóriada Conquista 45083-300, Brazil; danielaribeirodacosta@yahoo.com.br (D.R.d.C.); suzanyleite@yahoo.com.br (S.A.L.); mateus.santos.0712@gmail.com (M.P.d.S.); biacoelho20099@hotmail.com (B.S.C.); aldenise.moreira@gmail.com (A.A.M.); 2 Entomology Laboratory, Embrapa Cotton, Campina Grande 58428-095, Brazil; carlos.domingues-silva@embrapa.br; 3 Institute of Biology, Federal University of Bahia, Salvador 40107-115, Brazil; ibravo@ufba.br

**Keywords:** chitosan, eggs, fruit flies, kaolin, luminosity

## Abstract

**Simple Summary:**

Among the main phytosanitary problems that affect the production and commercialization of fresh fruits, the occurrence of fruit flies (Diptera: Tephritidae) is one of the main obstacles. The control of these tephritids is mainly performed through the use of toxic baits. The use of mineral films and biomaterials may constitute a viable alternative in relation to the traditional insecticide method, mainly because they do not contaminate the environment and do not leave toxic residues harmful to humans and animals in treated products. Therefore, by modifying the color and texture of the fruit cuticule that covers the plant tissues, kaolin affects the perception of arthropod pests, impairing the localization process and acceptance of the host plant and, consequently, its feeding and oviposition. In this study, we hypothesized that the color changes of guava fruits because of mineral particle films and biomaterials can affect the oviposition of fruit flies. The results obtained are promising and show that mineral films and biomaterials interfering with the color of guavas inhibited the oviposition of *A. obliqua*. Therefore, they can be used to protect guava fruits from the damage caused by this pest.

**Abstract:**

*Anastrepha obliqua* (Macquart, 1835) is an important pest of tropical fruits, especially Anacardiaceae and Myrtaceae, in the Americas. The objective of this study was to evaluate the influence of mineral films and biomaterials on the coloring of guava fruits (*Psidium guajava* L.) and implications for the oviposition of *A. obliqua*. Before the bioassays, color, firmness characteristics, total soluble solids, pH, and titratable acidity were determined to characterize the maturation stage of the fruits. Pieces of guava fruit covered in aluminum foil were immersed in suspensions of mineral particles (Surround^®^ WP kaolin; kaolins 605, 607, 608, and 611; and talc) and biomaterials (chitosan, cassava and potato starch, and guar gum) and distilled water (control). After drying, the fruits were exposed to two *A. obliqua* pairs for 48 h in choice and non-choice tests, and the numbers of eggs per fruit were counted. Mineral films (Surround^®^ WP kaolin, and kaolins 605, 607, 608, and 611) and biomaterials (cassava and potato starch) interfered with the color of guava (luminosity, chroma, and hue angle), inhibiting the oviposition of *A. obliqua*. Talc, chitosan, and guar gum did not influence the oviposition of *A. obliqua* in guava.

## 1. Introduction

Brazil is the world’s largest red guava (*Psidium guajava* L.) producer, reaching 578,600 tons in 2019, of which 34% was exported [1,2]. Among the most cultivated guava varieties, “Paluma and Pedro Sato” have a dual aptitude, for consumption in natura and processing industries [3].

The valorization of guava trees as raw material for the food industry and the increased consumption of in natura fruit are proportional to changes in the production system and commercialization. This is particularly true concerning the quality of the fruits produced, which can be affected by phytosanitary problems [4].

Guava is one of the fruits most affected by fruit flies (Diptera: Tephritidae) in Brazil [5]. Fruit fly larvae cause serious damage to fruit growth because they feed on the fruit pulp, making the fruit unsuitable for consumption in natura or industrialization [6]. Several factors, such as climate, altitude, geographical location, hosts, and adjacent orchards, can influence the diversity and dominance of fruit fly species in orchards [7]. Among these species, *Anastrepha obliqua* (Macquart, 1835) is an important pest of tropical fruits in the Americas, with great genetic variability among its populations and a wide geographical distribution, from northern Mexico to southeastern Brazil [8]. The most common hosts of *A. obliqua* are fruits of the family Anacardiaceae, such as the mango (*Mangifera indica* L.), the genus *Spondias* [9,10], and within the Myrtaceae family, mainly fruits of guava [11]. *Anastrepha obliqua* reach the peak of oviposition between 15 and 25 days, producing an average of 137 eggs per female, depositing one egg per oviposition [12,13].

To locate the host plant, female fruit flies can select oviposition sites based on the host plant species, size, color, odor, flavor, and maturation stage of the fruits, and avoid fruits previously oviposited [14]. Chemical stimuli, nutritional and inhibitory substances, or food stimulants also affect resource localization [15]. Fruit flies respond negatively to visual stimuli with high reflectance and wavelengths less than 520 nm, reducing oviposition and the capture of adults in traps [16,17,18].

The population suppression of fruit flies via behavioral manipulation using toxic baits (a mixture of attractive food and lethal agents) has become an important component of integrated pest management (IPM) programs worldwide [19,20,21,22,23,24,25,26,27]. However, the intensive use of toxic baits, such as the insecticide spinosad, can cause serious biological imbalances in fruit orchards by selecting resistant populations of this pest [28]. In addition, spinosad could also affect useful Arthropodofauna [29]. Thus, chemical insecticides are being used less to manage this pest, mainly because of pressure from consumers who prefer fresh fruits without residues, making it necessary to evaluate alternative strategies to manage this pest [30].

Mineral kaolin particle films and biomaterials are viable options for use in the replacement of synthetic chemical insecticides to avoid environmental contamination and the spread of toxic residues to humans and animals in the treated products [31,32].

Kaolin is an aluminosilicate mineral that is chemically inert, white, and formulated for use in plants [33]. The mechanisms of action of kaolin against insect pests include repellent, tactile, or visual interference, committed or interrupted oviposition and feeding activity, and decreased longevity and survival [34]. Therefore, by modifying the color and texture of the fruit cuticule that covers the plant tissues, kaolin affects the perception of arthropod pests, impairing the localization process and acceptance of the host plant and, consequently, its feeding and oviposition [35,36,37]. Unlike traditional agricultural chemicals, mineral kaolin particle films are inert and have no biochemical or physiological effects on plants or arthropod pests [38]. Thus, kaolin used in isolation does not cause fruit fly mortality [39,40], affect fruit fly attachment capacity on substrates treated with kaolin, or interfere with female oviposition behavior [41]; however, it can interfere with oviposition behavior [42]. When associated with entomopathogenic fungi, this product can cause insect pest mortality [43].

In addition to kaolin, biomaterial-based particle films have been used to protect cultivated plants because of their high availability, biodegradability and biocompatibility, and low toxicity [44,45]. In agriculture, these biomaterials are used mainly for the coating and preservation of fruits before and after harvest [46,47]. Cellulose, agar, starch, pectin, guar gum, alginates, carrageenan, xanthan gum, chitin, and chitosan are among the most commonly used natural polymers [47]. For example, chitosan is used to treat seeds, stimulate plant growth, and control phytopathogens [46,48]. When encapsulated in nanoparticles, chitosan is released gradually [46,47,49,50]. Chitosan also delays the fruit ripening process and inhibits the development of eggs and larvae of the *Anastrepha ludens* (Loew) [51,52].

Particle films based on minerals and biomaterials have been studied as important tools for the management of fruit flies in apples [53,54], nectarines [31,53], cherries [42], blueberries [40], citrus and peaches [31], and grapes [55]. Therefore, we hypothesized that the color changes of guava fruits, because of mineral particle films and biomaterials, can affect the oviposition of fruit flies, reducing their infestation in the field.

The objective of the present study was to evaluate the influence of mineral particles and biomaterial films on the coloring of guava fruits and their implications for the oviposition of *A. obliqua* in the laboratory.

## 2. Material and Methods

### 2.1. Origin of Anastrepha Obliqua and Fruits Used in Bioassays

Adults of *A. obliqua* fruit flies were obtained from Embrapa Mandioca and Fruticultura and maintained in an air-conditioned room of the Entomology Laboratory at the State University of Southwest Bahia in acrylic cages (30 × 30 × 30 cm). They were fed daily with a Bionis-based diet^®^, sugar (proportion 1:3) [56] and water and maintained at 25 ± 2 °C and 70 ± 10% relative humidity. Guava fruits of the Pedro Sato variety were offered to adult *A. obliqua* every two days for oviposition, and posteriorly removed and placed in plastic trays containing vermiculite to obtain larvae and pupae. The pupae were placed in 500 mL plastic pots containing a thin layer of vermiculite covered with paper towels until adult emergence.

The guava fruits (*Psidium guajava* L.) Pedro Sato variety with red colored pulp were obtained from the local fresh fruit trade and selected at maturation stage 2, based on the description by Azzolini et al. [57]. The use of guava fruits with red pulp in the present *A. obliqua* oviposition study facilitated the visualization of eggs and minimized possible experimental errors because of the contrast of the white color of the eggs of *A. obliqua* compared to the red color of the guava pulp.

Fruits were selected based on the light green color of the epicarp (peel), color uniformity, hue angle (between 116 and 113 h), and absence of oviposition orifices of fruit flies. The guavas were washed with 1% hypochlorite and cut in the part median, in average into 2 × 2 × 1 cm pieces (length, width, and height, respectively) (6 pieces). Based on the methodology described by Joachim-Bravo et al. [58], the pieces of guava were packaged in aluminum foil, such that only the peels were exposed for oviposition, and they were subsequently used in bioassays.

Before starting the bioassays, the physicochemical characteristics of the guava fruits, including firmness, color, total soluble solids (TSS), pH, and titratable acidity (TA), were determined to characterize their ripening stage. Firmness was evaluated using a penetrometer (model WA68, Italy) with an 8 mm diameter tip. Two readings were taken per fruit on opposite sides in the equatorial region, on 20 fruits, with results expressed in Newtons. The TSS content was determined by direct readings on a digital refractometer (Reichert, model r^2^ mini, Porto, Portugal); the results were expressed in °Brix, and the TA was determined by titrimetry [59], with results expressed as the % of citric acid per 100 g of pulp. The pH of 100 mL of guava juice was determined by direct readings using a digital potentiometer (Mars, model MB-10, São Paulo).

The color of the guava was determined previously and after applying the treatments on each piece of fruit, immediately after drying, using a colorimeter (CR-400, Minolta, Osaka, Japan). The apparatus was calibrated on a white ceramic plate using a D65 illuminant (z = 85.7; x = 0.3175; y = 0.3253). The luminosity values (L) were determined, which varied from 0 to 100 (black/white) and intensities of red/green (+/- (a) and yellow/blue (+/­) (b). Additionally, the color parameters were estimated as chroma C = (a^2^ + b^2^) 1/2, which represents the color purity, and the hue angle (Hue) H = tg^−1^ (b/a), which represents the color tone [40].

### 2.2. Oviposition: Non-Choice Tests

Two non-choice tests were performed to evaluate the effect of fruit acceptance of treated guava pieces as oviposition substrates. A completely random design was used with 11 treatments and four repetitions, evaluated on three consecutive days (one repetition every 48 h). Each non-choice test was performed using either a 100 or 200 g L^−1^ concentration of the tested mineral particle films or biomaterials. The treatments were as follows: T1, Surround^®^ WP kaolin; T2, kaolin 605 white; T3, kaolin 607 cream; T4, kaolin 608 white; T5, kaolin 611 grey; T6, talc 657; T7, chitosan; T8, cassava starch; T9, potato starch; T10, guar gum; and T11, control (distilled water). The particle films were dispersed in distilled water at concentrations of 100 and 200 g L^−1^ and guar gum was added to these suspensions at 5 g L^−1^, guar gum was used because it improves the viscosity and stability of formulations [60,61] except in the treatment T11 (control). These two concentrations were used because in preliminary tests with lower concentrations there was no verified effect on oviposition by the fruit fly. In the treatment with guar gum at 200 g L^−1^, the concentration of this substance in distilled water was also doubled (10 g L^−1^) to verify the effects of increasing the concentration.

Chitosan was obtained from the shells of crustaceans, dissolved in distilled water, and maintained under agitation for 2 min. Surround^®^ WP kaolin was obtained from NovaSource (Phoenix, AZ, USA), and kaolins 605, 607, 608, and 611, and talc were acquired from Brasilminas (Guarulhos, SP, Brazil). Biomaterial particle films were obtained from a natural product market (Indianópolis, SP, Brazil).

The bioassays were performed in the laboratory at 25 ± 2 °C and 70% relative humidity, with a 12 h photophase. The plot consisted of a plastic cage with a capacity of 3.5 L, containing a piece of treated guava and two pairs of 15-day-old naive *A. obliqua*, with 8 females per treatment, totaling 88 females. The pieces of guava were individually immersed for 10 s in 60 mL of each solution in a beaker. After immersion, the guava pieces were dried at 25 ± 2 °C for 1 h. Subsequently, a piece of guava was randomly selected and exposed to the fruit flies for 48 h in each cage over a disposable plastic cup with a capacity of 50 mL and subsequently removed to determine the number of eggs.

### 2.3. Oviposition: Choice Tests

The bioassay of choice was developed with an experimental design similar to that described in the previous section, with 10 combined treatments and 8 females per treatment, totaling 80 females/replica and 240 females in total (3 replicates). The difference was that in this bioassay, two pieces of guava were offered to the fruit flies by cage: one was treated with mineral film or biomaterial film, and the other was untreated and immersed in distilled water (control).

The methodology was the same as described in the previous bioassay, except for the control offered to the fruit flies jointly with the other treatments. The mineral particle films and biomaterials were mixed in distilled water at a concentration of 100 g L^−1^ and 200 g L^−1^, respectively. Guar gum was added to all treatments at a concentration of 5 g L^−1^, except for 200 g L^−1^, in which guar gum was used at a concentration of 10 g L^−1^. After immersion and drying, the pieces of guava (treated and untreated (control)) were separated by 10 cm and placed on plastic cups with a 50 mL capacity, in the lower part of each cage, containing one pair of fruit flies.

### 2.4. Statistical Analyses

The oviposition data of the non-choice test and color of the fruits (luminosity, chroma, and hue angle) were subjected to Bartlett and Shapiro–Wilk tests to evaluate the presence of homoscedasticity of variances of the treatments and the normality of the residues, respectively. When these assumptions were violated, the hue angle data after applying 100 and 200 g L^−1^ treatments and the number of eggs were transformed by $\sqrt{x}+1.$ Then, the data were compared using general linear models in the R software package “nlme” [62] and “lsmeans” [63]. A paired t-test was used to compare the average values of luminosity, chroma, and hue angle before and after applying the suspensions of 100 and 200 g L^−1^ [64].

The oviposition data obtained in the choice tests did not fit the assumptions of the analysis of variance, making it necessary to utilize randomization-type Monte Carlo simulations, with thousands of simulations to guarantee a 95% probability. To verify differences between treatments, a priori orthogonal contrasts were performed using R version 3.6.1 [64].

## 3. Results

### 3.1. Fruit Characterization

Before immersion in the treatments, guavas presented average values of TSS, TA, and pH were 7.0 ± 0.17 °Brix, 0.52 ± 0.01, and 3.40 ± 0.52, respectively. The average firmness of guava pulp was 45 ± 0.91 N. The color of the guavas before treatments at a concentrations of 100 g L^−1^ differed only in the chroma parameter (F = 82.101; df = 10, 43; *p* < 0.001), ranging from 37.73 ± 1.82 (kaolin 607) to 40.01 ± 0.32 (Surround^®^ WP kaolin); however, they did not differ from the control. The luminosity (F = 1.7272; df = 10, 43; *p* = 0.11583) and color angle (F = 1.2427; d f= 10, 43; *p* = 0.3017) did not differ between treatments (Table 1).

Film suspensions at 100 g L^−1^ affected the luminosity (t = 11.454; df = 43; *p* < 0.001), chroma (t = 9.9953; df = 43; *p* < 0.001), and hue angle (t = −8.0453; df = 39; *p* < 0.001). A comparison of the luminosity values before and after immersion in the 100 g L^−1^ suspension showed that all films increased the luminosity and hue angle, with a decrease in the chroma of the fruits, indicating immersion in mineral films and biomaterials influenced the change of guavas color (Table 1).

Differences were observed between treatments in luminosity (F = 49.405; df = 10, 43; *p* < 0.001), chroma (F = 480.53; df = 10, 43; *p* < 0.001), and hue angle (F = 187.934; df = 10, 43; *p* < 0.001) (Table 1) after immersion in 100 g L^−1^ suspensions. The luminosity and hue angles of the guava fruits before immersion in the suspensions were consistently lower than those after immersion in all treatments. Luminosity varied from 0 (black) to 100 (white), and the guavas after treatments had values between 55.77 ± 2.06 and 86.55 ± 1.73. The highest luminosities were observed in the fruits treated with Surround^®^ WP kaolin and kaolin 605, and the lowest was in the fruits treated with distilled water, followed by guar gum. In contrast, the largest hue angle was observed in fruits treated with kaolin 607, and the smallest was in those treated with distilled water and guar gum, with values ranging from 112 ± 0.82 to 152.75 ± 0.5.

Except for the control and guar gum, the chroma or purity of the color of the guava fruits before immersion in the suspensions was always lower than those after immersion in all treatments, with values ranging from 2.73 ± 0.18 to 40.63 ± 0.89 (Table 1). The highest chroma values were observed in fruits with treatments of guar gum and the control, and the lowest was in treatments with Surround^®^ WP kaolin and kaolins 605 and 608.

Guavas immersed in the 200 g L^−1^ suspension differed in luminosity (t = −11.293; df = 43; *p* < 0.001), chroma (t = 13.794; df = 43; *p* < 0.001), and hue angle (t = 235.42; df = 43; *p* < 0.001) (Table 2), compared to guavas before immersion (Table 2). The color values of the guavas after immersion at 200 g L^−1^ were different from those of guavas before immersion in the suspensions, demonstrating that all films modified this parameter.

There were no differences in luminosity (F = 1.4729; df = 10, 43; *p* = 19.36), chroma (F = 2.0251; df = 10, 43; *p* = 0.6254), or hue angle (F = 0.53799; df = 10, 43; *p* = 0.85047) in guava fruits before immersion in 200 g L^−1^ suspensions (Table 2). However, differences in luminosity (F = 1.4729; df = 10, 43; *p* = 19.36), chroma (F = 1.4729; df = 10, 43; *p* = 19.36), and hue angle (F = 1.4729; df = 10, 43; *p* = 19.36) (Table 2) were observed in fruits after immersion. The highest luminosities and lowest chroma of the guava fruits after immersion in the suspensions were observed in the Surround^®^ WP kaolin and kaolin 605 treatments, respectively. However, the lowest luminosities and the highest chroma were observed in fruits treated with distilled water and guar gum, respectively. The major hue angle was observed in fruits treated with kaolin 607 and the smallest in those treated with Surround^®^ WP kaolin, with values of 154.84 ± 1.49 (kaolin 607) and 98.44 ± 4.02 (Surround^®^ WP kaolin).

The luminosities of the fruits immersed in the 200 g L^−1^ suspensions were always greater than those of the fruits immersed in the 100 g L^−1^ suspensions (t = 4.9029; df = 43; *p* < 0.0001), except for chitosan (Table 1 and Table 2).

### 3.2. Oviposition: Non-Choice Tests

The number of eggs deposited by *A. obliqua* females in the pieces of guava immersed in the 100 g L^−1^ (AIC = 120.38; df = 43) and 200 g L^−1^ suspensions (AIC = 112.7; df = 43) varied between treatments in the non-choice test (Table 3). A small number of eggs were deposited by females of *A. obliqua* in the pieces of fruit treated with Surround^®^ WP kaolin and kaolin 608 at 100 g L^−1^ concentration and the highest were in those treated with chitosan at the same concentration.

However, in the 200 g L^−1^ concentration, a small number of eggs was deposited by *A. obliqua* females into pieces of fruit treated with Surround^®^ WP kaolin; kaolins 605, 607, 608, and 611; potato starch; and talc. The largest was for that treated with distilled water.

### 3.3. Oviposition: Choice Tests

**Figure 1:**
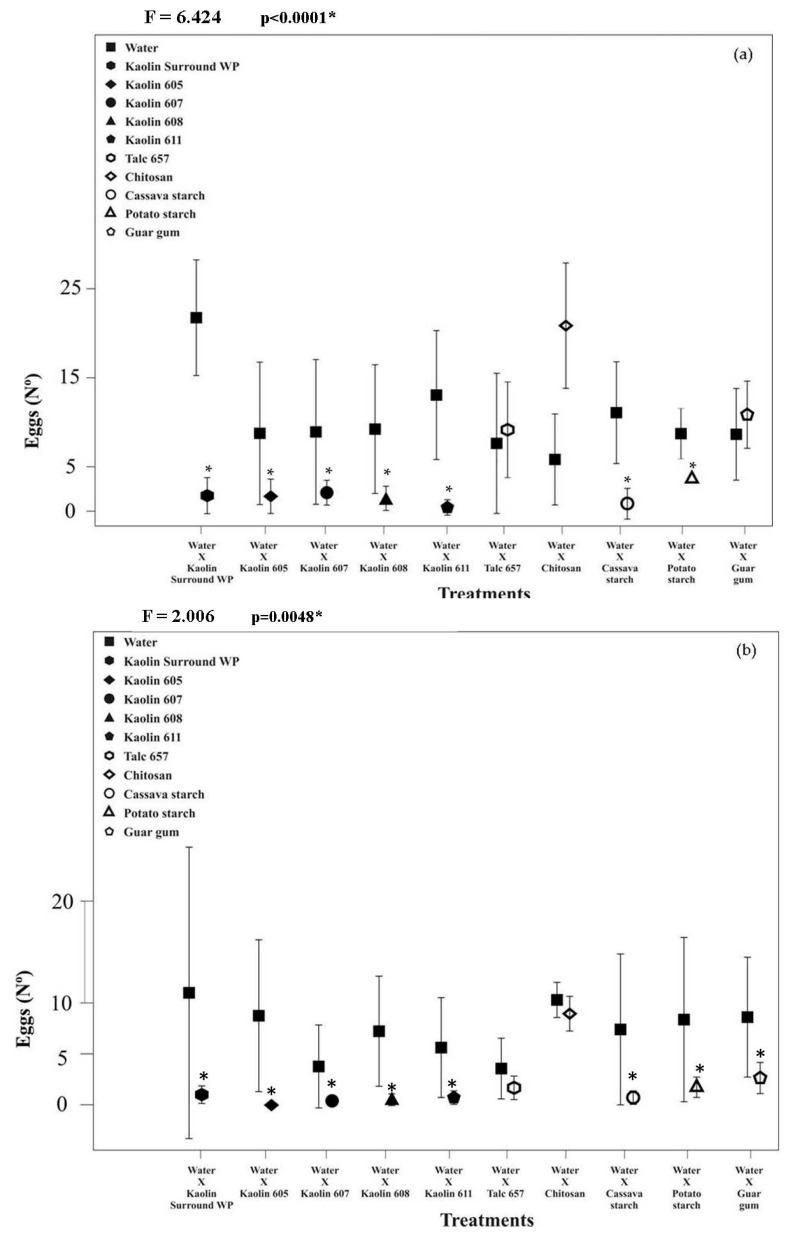
Number (N°) of *A. obliqua* eggs (mean ± standard deviation) in guavas, submitted the suspensions mineral and biomaterials at 100 g L^−1^ (**a**) and 200 g L^−1^ (**b**). Four repetitions per treatment were used.

In the choice bioassays, the number of eggs deposited by *A. obliqua* females in pieces of guava immersed in concentrations of 100 g L^−1^ (F = 6.424; df = 10; *p* < 0.0001) and 200 g L^−1^ (F = 2.006; df = 10; *p* = 0.048) varied between treatments (Figure 1).

Except for fruits treated with talc and chitosan at 100 g L^−1^, guar gum at 5 g L^−1^ (Figure 1a), and those treated with chitosan at 200 g L^−1^ (Figure 1b), a small number of postures of *A. obliqua* occurred in the other treatments with films of mineral particles of kaolin and biomaterials based on potato and cassava starch (Figure 1a). Talc applied at a 200 g L^−1^ concentration decreased the number of eggs deposited by *A. obliqua* females in the guava pieces. However, the observed variations in the standard deviation of the means were consistent with the small numbers of eggs deposited by *A. obliqua* in fruits treated with Surround^®^ WP kaolin, kaolin 611, cassava, and potato starch at 100 g L^−1^ concentration and only those treated with kaolins 605 and 608 at a concentration of 200 g L^−1^ of.

## 4. Discussion

The similarity in luminosity and hue angle of the peel between the guava fruits used in the bioassays before applying the suspensions of mineral particle films and biomaterials confirmed that they were in a similar stage of maturation, with small variations in chroma (Table 1). These results corroborate those obtained by Azzolini et al. [57], who characterized maturity stage 2. This is important because the insertion of the aculeus of the flies in the fruits depends on several factors, including the type of host (primary or secondary), evidence of previous use by conspecifics (presence of pheromone marking), and quality of the fruit (i.e., degree maturation) [15]. Visual and tactile stimuli influence the recognition and acceptance of fruit as places of oviposition, making it difficult to location of oviposition sites and/ or the fixation of females on coated fruits [41]. In present study, the reduction in the oviposition of *A. obliqua* may not have been caused by the difficulty in locating the fruit due to the color change (visual stimulus) and the change in the texture of the skin due to the presence of the films (tactile stimulus).

The small number of eggs deposited by *A. obliqua* females in the pieces of fruit treated with Surround^®^ WP kaolin and kaolin 608 at a 100 g L^−1^ concentration and in those treated with Surround^®^ WP kaolin; kaolins 605, 607, 608, and 611; and potato starch and talc at 200 g L^−1^ in the non-choice test indicated that the mineral particle films used at the minor concentration were more suitable for protecting guava fruits than those of biomaterials. These results corroborate those of studies on kaolin applications that inhibited the oviposition of *C. capitata* in apples [54] and citrus fruits [31] at a concentration of 30 g L^−1^ in the laboratory and with those conducted in citrus orchards [32,65] and apples [66] sprayed with 50 g L^−1^ Surround^®^ kaolin. However, the increase in the number of treatments with fewer postures of *A. obliqua*, both for mineral particles and for biomaterials in the fruits treated at a concentration of 200 g L^−1^ can be attributed to the uniform coating of the fruits provided by the higher concentration of these products [67].

In the non-choice test, when the treated and untreated fruits were offered simultaneously to laying *A. obliqua* females, an effect of the mineral particles and biomaterial films was observed regardless of concentration (100 g L^−1^ or 200 g L^−1^). All mineral films and biomaterials based on potato and cassava starch and guar gum reduced *A. obliqua* oviposition. The preference of some tefrithids for certain colors depends on both color tone (chroma) and the intensity of the total reflected light (luminosity) [68]. For example, *A. obliqua* is attracted by wavelengths ranging from 340 nm to 670 nm, with a peak of attraction between 380 and 570 nm, corresponding to the electromagnetic spectrum where ultraviolet and visible light occur [18]. Therefore, the change of the natural green color of the guava fruit peel to the white color of the films of mineral particles or biomaterials probably impaired the perception of the *A. obliqua* females. Studies have shown that fruits or spheres covered with white coating reduce the oviposition of fruit flies [16,18,68]. The white color has a high reflectance and is less visually attractive to fruit flies, as demonstrated for *C. capitata* [68,69], *Bactrocera dorsalis* (Hendel) [70], and *A. obliqua* [18].

In general, it was verified that the 200 g L^−1^ suspension inhibited oviposition in choice and non-choice tests. Inhibition of oviposition of *C. capitata* was also obtained with the use of kaolin (Inducal^®^) and calcareous liquid, applied at the same concentration, in apple and mango fruits [71]. However, it was observed that 50% of the particle film-based biomaterials in the choice and non-choice tests did not protect the fruits from oviposition by *A. obliqua*. The exceptions were for potato starch, applied at a concentration of 200 g L^−1^, which reduced the oviposition of flies in the bioassays of choice and non-choice, and cassava starch in the choice bioassay at the two concentrations tested. Several studies have been conducted with particle films based on edible biomaterials, such as starches, for post-harvest protection of fruits [72,73,74,75].

In the present study, potato and cassava starches were demonstrated to be promising for the protection of guava fruits because, in addition to preserving the color of the peel, they protected the fruit pulp from *A. obliqua* oviposition after 48 h of exposure to the insects. However, further studies in the laboratory and field should be conducted because with increased concentrations, the starch base films became brittle, exposing the fruit to flies. This is a common result, particularly in treatments with higher concentrations of this product [74,75].

The chitosan base film did not differ from the control in both bioassays for the number of eggs deposited by *A. obliqua*. This was because the product formed a semitransparent film, which delayed the ripening of the guava fruits and maintained them at the same color as the maturation stage 2 peel, similar to that of the control fruits. The maintenance of peel integrity and delaying the ripening of guava fruits are effects of chitosan, as observed by Hong et al. [76]. When applied to grapes, chitosan did not inhibit *C. capitata* but stimulated oviposition by this fruit fly [54]. Studies conducted after oviposition revealed that chitosan inhibited the development of eggs and larvae of *A. ludens* and *A. obliqua* in mangos [52,77].

Guar gum added to all suspensions of mineral particles and biomaterial films did not affect the oviposition of *A. obliqua*, except in the choice bioassay, when it was used at 10 g L^−1^. Guar gum acts as a thickener, improving the viscosity and stability of formulations, and is commonly used in chemical and biological insecticide formulations [60,61] and as a diet for the mass production of the fruit flies and parasitoids [78]. In a similar study, guar gum, when used as a thickener in suspensions of mineral films and biomaterials, did not affect the inhibition of oviposition by *C. capitata* [55].

## 5. Conclusions

The results obtained in the present study are promising and show that mineral films (Surround^®^ kaolin, and kaolins 605, 607, 608, and 611) and biomaterials (cassava and potato starch) changed the color of guavas (luminosity, chroma, and hue angle), inhibiting the oviposition of *A. obliqua*. Therefore, they can be used to protect guava fruits from the damage caused by this pest. Additionally, different species of fruit flies vary their oviposition behavior in fruits treated with the studied particles. New studies should test films of mineral particles and biomaterials in other hosts for females of species of economic importance, since the oviposition behavior of fruit flies is probably regulated by an interaction of factors. Finally, it demonstrates the potential of biomaterials to protect fruits against attack by fruit flies, mainly because they are edible and rapidly degrade.

## Figures and Tables

**Table 1:** Luminosity, chroma and hue angle (mean ± standard deviation) of the guavas before and after immersion in suspensions at 100 g L^−1^.

Treatments	Before Immersion in Suspension at 100 g L^−1^			After Immersion in Suspension at 100 g L^−1^		
	Luminosity	Chroma	Hue Angle	Luminosity	Chroma	Hue Angle
T1-Kaolin Surround^®^ WP	54.71 ± 0.12 a	40.01 ± 0.32 a	113.78 ± 1.11 a	86.55 ± 1.73 a	2.87 ± 0.07 e	123.00 ± 0.0 e
T2-Kaolin 605 white	55.94 ± 1.15 a	39.01 ± 0.63 ab	114.32 ± 1.70 a	83.39 ± 1.72 a	3.45 ± 0.38 e	138.25 ± 2.63 bc
T3-Kaolin 607 cream	53.86 ± 1.91 a	37.73 ± 1.82 b	114.17 ± 1.00 a	74.12 ± 2.36 b	20.40 ± 1.61 c	152.75 ± 0.5 a
T4- Kaolin 608 white	55.05 ± 1.01 a	38.36 ± 0.42 ab	115.61 ± 2.67 a	70.41 ± 4.80 bc	2.73 ± 0.18 e	126.75 ± 5.62 de
T5- Kaolin 611 grey	53.04 ± 1.35 a	37.96 ± 0.47 ab	114.25 ± 0.95 a	70.99 ± 3.00 bc	13.80 ± 1.03 d	143.5 ± 1.91 b
T6-Talc 657	56.14 ± 1.52 a	38.36 ± 1.32 ab	116.45 ± 1.31 a	73.42 ± 2.25 b	11.59 ± 1.41 d	137.25 ± 2.36 c
T7-Chitosan	55.44 ± 1.54 a	39.09 ± 0.60 ab	115.10 ± 1.16 a	64.69 ± 0.98 cd	28.41 ± 1.38 b	124.75 ± 4.03 de
T8-Cassava starch	56.13 ± 2.10 a	39.41 ± 0.55 ab	115.51 ± 1.68 a	68.71 ± 3.51 bcd	22.19 ± 1.37 c	129.75 ± 0.96 d
T9-Potato starch	55.70 ± 1.98 a	39.53 ± 1.27 ab	114.06 ± 1.96 a	62.73 ± 2.83 de	30.12 ± 1.85 b	136.25 ± 2.87 c
T10-Guar gum	54.08 ± 1.78 a	39.08 ± 1.44 ab	113.69 ± 1.68 a	58.01 ± 2.61 ef	40.63 ± 0.89 a	112.00 ± 0.82 f
T11-Distilled water	55.74 ± 1.77 a	39.70 ± 0.4 ab	114.94 ± 1.33 a	55.77 ± 2.06 f	40.20 ± 2.08 a	112,25 ± 1.70 f
CoefficientVariation (%)	2.86	2.5	1.37	3.89	6.54	2.05

Means followed by the same lowercase letter in the column are not different by the Tukey test (*p* < 0.05). Four repetitions per treatment were used.

**Table 2:** Luminosity, chroma and hue angle (mean ± standard deviation) of the guavas before and after immersion in suspensions at 200 g L^−1^.

Treatments	Before Immersion in Suspension at 200 g L^−1^			After Immersion in Suspension at 200 g L^−1^		
	Luminosity	Chroma	Hue Angle	Luminosity	Chroma	Hue Angle
T1- Kaolin Surround^®^ WP	53.60 ± 5.3 a	40.07 ± 2.09 a	113.77 ± 2.40 a	91.08 ± 2.98 a	3.52 ± 0.21 h	98.44 ± 4.02 d
T2- Kaolin 605 white	52.96 ± 6.38 a	41.86 ± 1.87 a	114.34 ± 2.50 a	91.18 ± 0.75 a	4.57 ± 0.52 gh	106.27 ± 10.18 cd
T3- Kaolin 607 cream	54.52 ± 4.24 a	39.58 ± 1.78 a	116.95 ± 3.29 a	79.59 ± 4.26 bc	14.09 ± 0.94 d	154.84 ± 1.49 a
T4- Kaolin 608 white	55.19 ± 3.68 a	43.13 ± 1.29 a	116.44 ± 4.57 a	72.69 ± 1.75 c	6.24 ± 0.68 efg	134.09 ± 1.01 b
T5- Kaolin 611 grey	49.63 ± 3.39 a	39.14 ± 3.57 a	114.06 ± 2.41 a	75.47 ± 2.12 c	7.98 ± 0.40 e	133.04 ± 1.22 b
T6- Talc 657	49.72 ± 4.80 a	39.34 ± 3.66 a	116.26 ± 5.07 a	84.60 ± 1.68 ab	6.92 ± 0.23 ef	127.05 ± 2.25 b
T7- Chitosan	55.86 ± 2.71 a	41.95 ± 1.71 a	114.48 ± 2.14 a	58.07 ± 1.86 d	18.95 ± 0.98 c	110.94 ± 2.61 cd
T8- Cassava starch	58.62 ± 2.34 a	42.96 ± 1.10 a	116.85 ± 1.98 a	79.79 ± 1.23 bc	5.49 ± 0.30 fg	110.14 ± 4.36 cd
T9- Potato starch	57.28 ± 2.26 a	39.35 ± 1.49 a	116.76 ± 5.84 a	73.97 ± 3.82 c	7.08 ± 0.68 ef	106.36 ± 1.88 cd
T10- Guar gum	51.21 ± 2.21 a	40.90 ± 1.21 a	114.46 ± 2.59 a	57.47 ± 6.04 d	37.70 ± 1.10 b	114.14 ± 1.04 c
T11- Distilled water	51.69 ± 0.72 a	40.25 ± 0.41 a	114.86 ± 2.14 a	55.84 ± 2.84 d	39.68 ± 1.18 a	115.67 ± 2.57 c
CoefficientVariation (%)	7.18	5.09	2.97	4.09	5.34	3.26

Means followed by the same lowercase letter in the column are not different by the Tukey test (*p* < 0.05). Four repetitions per treatment were used.

**Table 3:** Estimates for GLM parameters with model Gaussian for the number of eggs (mean ± SE) of *A. obliqua* in guavas, subjected to suspensions at 100 and 200 g L^−1^ no-choice tests.

Treatments	Suspension at 100 g L^−1^				Suspension at 200 g L^−1^					
	Estimate	Error Standard	Z-Value	*p*-Value	Eggs (N°) ^1^	Estimate	Error Standard	Z-Value	*p*-Value	Eggs (N°) ^1^
(Intercept)	0.707	0.4177	0.0999	0.0999	-	−4.017	0.000	0.000	1.0000	-
T1-Kaolin Surround^®^ WP	-	-	-	-	0.70 ± 0.42 a	-	-	-	-	0.0 ± 0.38 a
T2- Kaolin 605 white	0.539	0.5907	0.3690	0.3682	1.25 ± 0.42 ab	3.231	0.000	0.597	0.5547	0.32 ± 0.38 a
T3- Kaolin 607 cream	0.323	0.5907	0.5884	0.5884	1.03 ± 0.42 ab	4.228	0.000	0.000	1.0000	0.0 ± 0.38 a
T4- Kaolin 608 white	0.161	0.5907	0.7863	0.7863	0.87 ± 0.42 a	1.436	0.000	0.265	0.7924	0.14 ± 0.38 a
T5- Kaolin 611 grey	0.730	0.5907	0.2249	0.2249	1.44 ±0.42 ab	3.677	0.000	0.000	1.0000	0.0 ± 0.38 a
T6- Talc 657	0.515	0.5907	0.3896	0.3896	1.22 ± 0.42 ab	−6.206	0.000	0.000	1.0000	0.0 ± 0.38 a
T7- Chitosan	2.109	0.5907	0.0011 **	0.0011 **	2.85 ± 0.42 b	5.590	0.000	1.033	0.3092	0.56 ± 0.38 ab
T8- Cassava starch	0.871	0.5907	0.1498	0.1499	1.58 ± 0.42 ab	5.403	0.000	0.998	0.3255	1.17 ± 0.38 ab
T9- Potato starch	1.840	0.5907	0.0038 **	0.0038 **	2.55 ± 0.42 b	2.046	0.000	0.378	0.7078	0.17 ± 0.38 a
T10- Guar gum	0.865	0.5907	0.1524	0.1524	1.57 ± 0.42 ab	1.500	0.000	2.771	0.0091 **	1.50 ± 0.38 b
T11- Distilled Water	1.677	0.5907	0.0077 **	0.0077 **	2.38 ± 0.42 b	2.175	0.000	4.017	0.0003 ***	2.17 ± 0.38 b
AIC					120.38					112.7

** *p* ≤ 0.01, *** *p* ≤ 0.001; ^1^ Data transformed in √x + 1. Mean ± SD values in the same column followed by the same letter do not differ significantly at *p* < 0.01.

## Data Availability

Data is contained within the article.
